# NLRC5: A Potential Target for Central Nervous System Disorders

**DOI:** 10.3389/fimmu.2021.704989

**Published:** 2021-06-18

**Authors:** Lu Zhang, Cui Jiao, Lingjuan Liu, Aiping Wang, Li Tang, Yi Ren, Peng Huang, Jie Xu, Dingan Mao, Liqun Liu

**Affiliations:** ^1^ Department of Pediatrics, The Second Xiangya Hospital, Central South University, Changsha, China; ^2^ Children’s Brain Development and Brain Injury Research Office, The Second Xiangya Hospital, Central South University, Changsha, China

**Keywords:** pattern recognition receptor, NLRC5, central nervous system, disease, development

## Abstract

Nucleotide oligomerization domain-like receptors (NLRs), a class of pattern recognition receptors, participate in the host’s first line of defense against invading pathogenic microorganisms. NLR family caspase recruitment domain containing 5 (NLRC5) is the largest member of the NLR family and has been shown to play an important role in inflammatory processes, angiogenesis, immunity, and apoptosis by regulating the nuclear factor-κB, type I interferon, and inflammasome signaling pathways, as well as the expression of major histocompatibility complex I genes. Recent studies have found that NLRC5 is also associated with neuronal development and central nervous system (CNS) diseases, such as CNS infection, cerebral ischemia/reperfusion injury, glioma, multiple sclerosis, and epilepsy. This review summarizes the research progress in the structure, expression, and biological characteristics of NLRC5 and its relationship with the CNS.

## Background

The innate immune response is the first line of defense against pathogen invasion. Pattern recognition receptors (PRRs) act as pivotal sensors in these processes by recognizing specific microbial pathogens through pathogen-associated molecular patterns (PAMPs) and damage-associated molecular patterns (DAMPs). PRRs induce intracellular cytokine and chemokine secretion, trigger an inflammatory response, and ultimately activate the host defense system (1). To date, biochemical studies have identified four types of PRR families: toll-like receptors (TLRs), retinoic acid-inducible gene-I-like receptors (RLRs), nucleotide oligomerization domain (NOD)-like receptors (NLRs), and C-type lectin receptors (CLRs) (2). NLRs are a large protein family of PRRs located in the cytoplasm, where they are involved in the activation of the inflammatory response system and rapid removal of invasive pathogens. It was previously reported that NLRC5 [NLR family caspase recruitment domain (CARD) containing 5, also known as NOD4, NOD27, and CLR16.1] accounts for a large proportion of the NLR family, which can modulate immune responses in the context of many human diseases, such as liver disease, renal disease, rheumatoid arthritis, and heart disease, by regulating nuclear factor-κB (NF-κB), type I interferon (IFN-1), inflammasome signaling pathways and the expression of the major histocompatibility complex (MHC) I genes (3–5). However, in recent years, studies have found that NLRC5 is also associated with CNS infection (CNSI) (6–9), neuronal development (10), and neuropsychiatric disorders, such as cerebral ischemia/reperfusion (I/R) injury (11, 12), glioma (13–15), multiple sclerosis (MS) (16), epilepsy (17), schizophrenia (SCZ), and bipolar disorder (BD) (18, 19). Following recent advances in this area, we focus on a comprehensive update on the structure, expression, and biological characteristics of NLRC5, as well as its relationship with the CNS.

## Characteristics of NLRC5

### NLRC5 Structure

**Figure 1:**
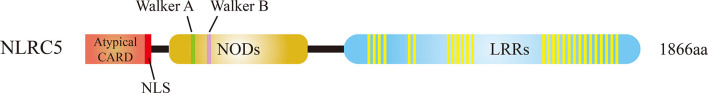
Schematic representation of NLRC5 structure CARD: Caspase recruitment domain; NOD: Nucleotide oligomerization domain; LRR: Leucine-rich repeats NLS: Nuclear localization signal; Walker A: Nucleoside triphosphate (NTP)-binding site, Walker B: NTP hydrolysis site.

To date, 23 NLR genes have been identified in the human genome, whereas there are at least 34 mouse NLR genes in the mouse genome, and they are widely expressed in various cells and tissues (1, 4). It has been found that tripartite domain architecture is a common feature of the structure of this protein family: a variable N-terminal domain that interacts with downstream corresponding protein molecules, NODs that lead to the oligomerization and activation of NLRs, and a C-terminal leucine-rich repeat (LRR) domain that recognizes ligands (such as PAMPs). In terms of the N-terminal domains, NLRs can be classified into three subfamilies: NLRC containing CARD, NLRP containing pyrin domain (PYD), and NAIP containing baculovirus inhibitor domain (BIR) (1). *NLRC5* was initially cloned in 2010 and has been confirmed to be located on human chromosome 16q13 (20, 21), which straddles a region of approximately 96 kbp. The encoded polypeptide contains 1,866 amino acids, with a predicted size of approximately 200 kDa (1). NLRC5 is similar to the tripartite domain of other NLRs (**Figure 1**). However, the N-terminal CARD of NLRC5 has been predicted to adopt a death domain fold and exhibits no obvious sequence similarity with other typical CARDs; therefore, it is called an atypical CARD (20, 22). In addition, the NOD of NLRC5 contains the Walker A and Walker B motifs, which are important for nucleoside triphosphate (NTP) binding and NTP hydrolysis, respectively (23). NLRC5 is the largest member of the NLR protein family because of the unusually long stretch of 27 LRRs in its C-terminal region (1, 20, 24). The structure of NLRC5 is highly conserved in many mammalian species; for example, the homology of human NLRC5 to mouse NLRC5 is 64% (20), which suggests that NLRC5 has similar crucial functions in various organisms.

### Expression of NLRC5

#### NLRC5 Expression in the Tissue

Previous studies have shown that NLRC5 predominantly exists in the bone marrow, human THP-1 cells, B cells, human cervical cancer cell lines, and primary myeloid and lymphoid cells (3). These locations indicate that NLRC5 plays a vital regulatory role in the occurrence and development of immune diseases. Although NLRs play a crucial role in innate immunity, there have been relatively few studies on their expression in the brain. Kuenzel et al. found that the highest expression of *NLRC5* mRNA in humans was in the tissues of the brain, lung, and prostate, followed by the heart, digestive tract, and thymus (25). Subsequently, researchers found that NLRC5 is expressed in both the brain and the cells forming the blood-brain barrier (BBB), including neurons, astrocytes, microglia, oligodendrocytes, endothelial cells, and brain pericytes (**Table 1**) (2, 26–28). Specifically, the mRNA and protein expression of NLRC5 is relatively abundant in the hippocampus of the mouse brain. Using qPCR and western blotting analysis, Li et al. identified that there is a gradual increase in expression from 0 to 15 days after birth (10). Thus, NLRC5 may be related to the physiological and pathological states of the CNS to some extent.

**Table 1:** NLRC5 expression in the tissue.

Protein	Expression	Location	Involvements	References
NLRC5/NLRC5 mRNA	Bone marrow	Cytoplasm/nucleus	23 NLR genes in human genome 34 NLR genes in mice genome	(3)
	THP-1 cells			
	B cells			
	Cervical cancer cell lines			
	Primary cells of myeloid			
	Primary cells of lymphoid sources			
	Brain(neurons, astrocytes, microglia, oligodendrocytes, endothelial cells, and brain pericytes)			(2, 26–28)
	Lung			(25)
	Prostate			
	Heart			
	Digestive tract			
	Thymus			

#### Intracellular NLRC5 Localization

Using immunofluorescence analysis, Kuenzel et al. found that NLRC5 is restricted to the cytoplasm to recognize intracellular danger signals (25). In contrast, Meissner et al. revealed that NLRC5 is located in both the cytoplasm and nuclear compartments (29). Moreover, NLRC5 has transcriptional regulation properties similar to those of the class II transactivator (CIITA), which may also be a nuclear localization protein molecule that can shuttle between the nucleus and the cytoplasm (3). NLRC5 was found to be more frequently localized to the cytoplasm of cells with high NLRC5 levels, whereas nuclear localization was more frequently observed in cells with low NLRC5 levels (1, 29). Accordingly, the nuclear localization of NLRC5 was not the result of overexpression. High expression of NLRC5 was observed in the nucleus of cells subjected to leptomycin B (an inhibitor of CRM1-dependent nuclear export) treatment (29), suggesting that the NLRC5 plasmid shuttle may be carried out in a CRM1-dependent manner. Furthermore, some studies have indicated that NLRC5 possesses a nuclear localization signal (NLS) between the CARD and NOD in duplicate, in which locus mutations within the NLS block the translocation of NLRC5 from the cytoplasm to the nucleus (29). Moreover, the CARD and NBD of NLRC5 are involved in its nuclear import because of the NLSs.

### Biological Function of NLRC5

#### NLRC5 Is a Negative Regulator of NF-κB

**Figure 2:**
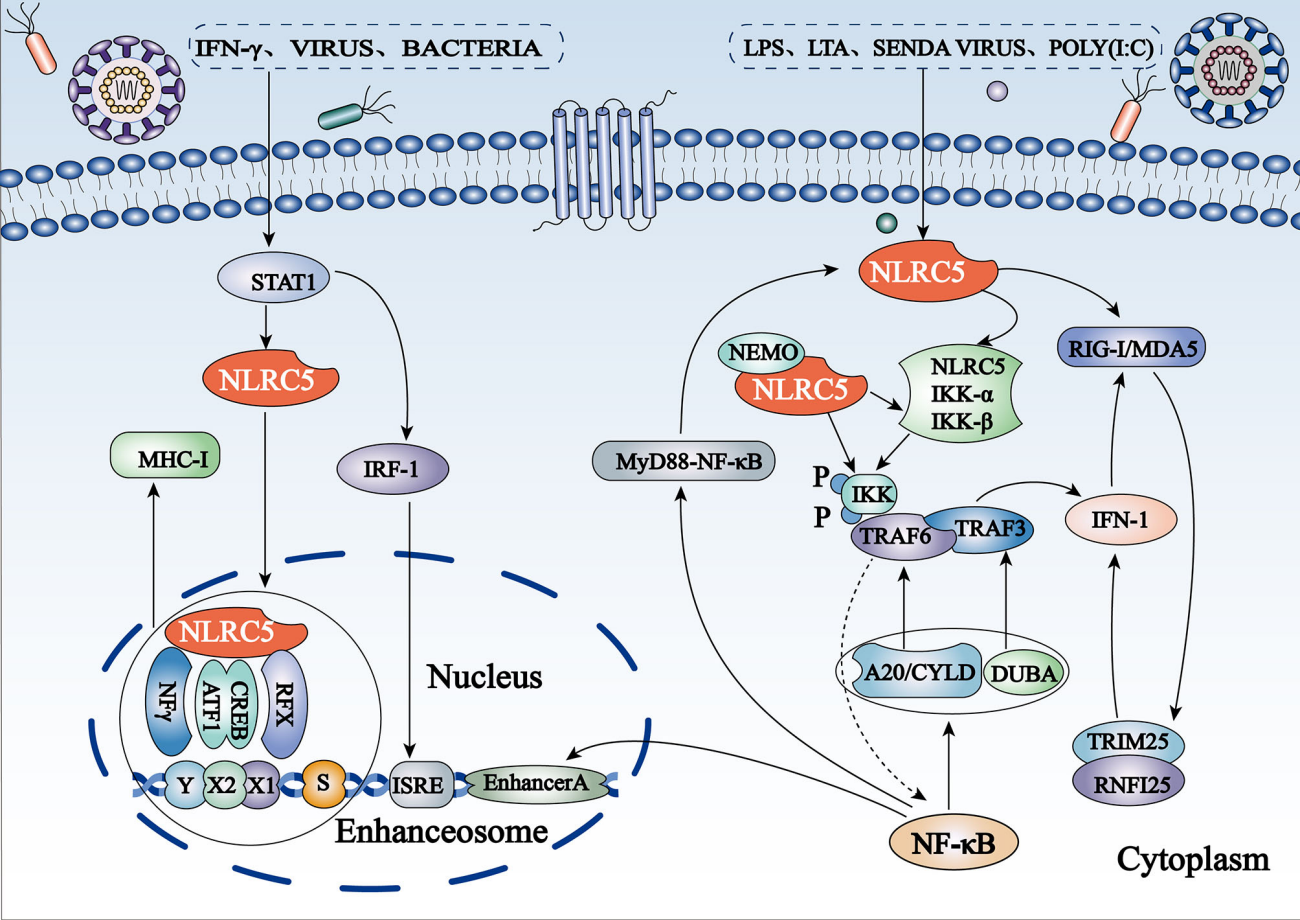
Schematic representation of regulation of the NF-κB and IFN-I pathways and MHC I gene expression by NLRC5 (1) NLRC5 inhibits the NF-κB pathway by binding to IKKα/IKKβ subunits and blocking their phosphorylation. The expression of NLRC5 itself is regulated by NF-κB activation to form a negative regulatory feedback loop. (2) NLRC5 can also suppress RIG-like receptor-mediated IFN-I responses by interacting with RIG-I/MDA5. (3) Stimuli such as IFN-γ, viral infection, and bacterial infection induce NLRC5 to bind directly to the GAS site in the NLRC5 promoter by activating the STAT1 homologous dimer. Subsequently, NLRC5 shuttles from the cytoplasm to the nucleus and combines with the S-X-Y motif to form a potent enhanceosome and to induce MHC I transcription.

NF-κB is ubiquitously expressed in mammalian cells and plays a critical role in innate and adaptive immunity. Dysregulation of NF-κB activity leads to a variety of diseases, such as immunodeficiency, viral infections, inflammation, and cancer (1). In addition, NF-κB in the CNS can be activated by growth factors (brain-derived neurotrophic factor and nerve growth factor) and excitatory neurotransmitters, such as glutamate. It is also involved in the regulation of synaptic plasticity, learning, and memory (30), as well as the development and maintenance of addiction to a stimulus (31). The IκB kinase (IKK) complex is composed of the kinase heterodimer IKKα/IKKβ and a regulatory subunit. Cui et al. confirmed for the first time that NLRC5 is a potent negative regulator of NF-κB activation through luciferase assays (32). NLRC5 competes with NF-κB essential modulator, an NF-κB essential modulator, to directly interact with IKKα/IKKβ subunits and to block their phosphorylation and kinase activity (**Figure 2**), which is related to the NLRC5 LRR domain (32). NLRC5 overexpression affects the translocation of p65 NF-κB nuclear translocation and inhibits the NF-κB downstream pathway, thereby playing an anti-inflammatory role (33). Nonetheless, how NLRC5 regulates the effects of the NF-κB signaling pathway on brain function requires further study.

#### NLRC5 Is a Negative Regulator of IFN-I

NLRC5 was initially reported to be a positive regulator of IFN-I signaling. Vesicular stomatitis virus (VSV), polyinosinic: polycytidylic acid [poly(I:C)], and lipopolysaccharides (LPS) can induce IFN-I signal transduction (32). Endogenous NLRC5 knockdown reduced Sendai virus and poly(I:C)-mediated IFN-I pathway-dependent responses in human primary dermal fibroblasts and THP-1 cells and significantly inhibited the secretion of IFN-γ in human fibroblast cells infected with cytomegalovirus (25, 34). In contrast, growing evidence has confirmed that NLRC5 is a negative regulator of IFN-I. *In vitro* experiments have shown that the secretion of IFN-β is significantly increased in *NLRC5* ^-/-^ macrophages exposed to RIG-I/MDA5 ligands, such as VSV and poly(I:C), suggesting that NLRC5 may mainly interact with RIG-I/MDA5 to inhibit RLR-mediated IFN-I responses (32, 35) (**Figure 2**). In addition, NLRC5 inhibits RLR-mediated IFN-I secretion in plasmacytoid dendritic cells (36). These phenomena have also been confirmed *in vivo*: NLRC5-deficient mice produce higher amounts of IFN-β in sera when challenged with LPS or infected with VSV (35).

#### NLRC5 Transactivates MHC Class I Genes

MHC I/II and co-molecules activate an adaptive immune response under many pathological conditions, such as autoimmunity, cancer, and infection, by presenting antigens to T lymphocytes. In 2010, Meissner et al. initially identified that NLRC5 may directly regulate the expression of MHC I genes (29). Gene-chip analysis showed significantly higher gene expression in wild-type and mutant Walker B cell lines, which express the active forms of NLRC5, compared to cells expressing the mutant Walker A and mutant Walker AB of NLRC5 (29). The most upregulated genes included the MHC I family (*HLA-A, -B, -C*, and *-E*) and those associated with antigen presentation and processing of MHC I genes (*β2M, LMP2*, and *TAP1*), both of which were induced by active NLRC5, which requires NTP binding instead of NTP hydrolysis (29). Given the lack of a DNA-binding domain, NLRC5-mediated MHC I transactivation requires the S-X-Y motifs in the proximal regions of MHC I genes, which are dependent on other components, called enhanceosomes, to connect to the promoter region (10). IFN-γ leads to binding of transcriptionally active signal transducers and activators of transcription-1 (STAT1) to cis-elements of the gamma interferon activation site (GAS) *via* stimulation of the JAK/STAT pathway to promote the upregulation of NLRC5 expression (23). NLRC5 shuttles from the cytoplasm to the nucleus and forms a potent enhanceosome with transcription factors, such as X1 box regulatory factor X (RFX), X2 box activating transcription (ATF)/cAMP response element binding protein (CREB), and nuclear factor-Y protein (NFY) complexes, thereby inducing activation of MHC I gene transcription (23) (**Figure 2**). Located at the enhanceosome complex, NLRC5 has been suggested to function as a scaffold to recruit transcriptional initiation and elongation factors, as well as co-activators [such as CREB binding protein (CBP), p300, GCN5, and PCAF] and chromatin modifiers (23). More intuitively, NLRC5 has been shown to be essential for the removal of the gene-silencing trimethylation of lysine 27 on histone 3 (H3K27me3) on the MHC I promoter (37), which is likely the result of specific recruitment of demethylating enzymes by NLRC5.

#### NLRC5 Activates Inflammasomes

Inflammasomes are multiprotein complexes that are assembled by PRRs in the cytoplasm. These complexes are a significant part of the innate immune system and are known for their ability to recognize PAMPs or DAMPs and activate the proteolytic enzyme caspase-1. Activated caspase-1 promotes proteolysis and maturation of the pro-inflammatory cytokines IL-1β and IL-18, stimulating the occurrence of pyroptosis (38). It has been reported that inflammasome activation is associated with a variety of brain diseases, including ischemic and traumatic brain injury and neurodegenerative diseases (12, 39, 40). Co-immunoprecipitation experiments revealed that NLRC5 directly binds to the NLR family PYD containing 3 (NLRP3) and apoptosis-associated speck-like protein containing CARD (ASC), which is considered to be an activator and synergetic component of the inflammasome (41). *In vitro* studies have shown that the overexpression of NLRC5 promotes IL-1β production by stimulating caspase-1 in HEK293 cells (42). Candidate pathogens such as *Escherichia coli*, *Shigella flexneri*, and *Staphylococcus aureus* require NLRP3 and ASC to activate the inflammasome, and RNA-mediated suppression of NLRC5 was shown to significantly block IL-1β secretion in THP-1 cells compared to the control group (41). *In vivo*, NLRC5 was shown to significantly promote IL-1β secretion in *Listeria monocytogenes*, and the recruitment of neutrophils and bacterial clearance were significantly reduced in the liver and spleen of *Nlrc5*-deficient mice (43). When attacked by pathogenic microorganisms or stimulated by endogenous danger signals, host cells produce caspase-1-dependent pyroptosis. Different from chromatin pyknosis, DNA fragmentation, cell shrinkage and apoptotic body formation in the process of apoptosis, pyroptosis will cause the infected cells to swell and rupture the cell membrane in a short period of time, thus releasing inflammatory factors such as IL-1 β and IL-18 into the interstitial space, activating the corresponding receptors in the adjacent cells and triggering a broader immune response (44, 45). These findings suggest that NLRC5 regulates inflammasome activation and participates in the formation of IL-1β in response to multiple bacterial pathogens. In macrophages, NLRC5 overexpression promotes caspase-1-dependent IL-1β production. However, NLRC5 deficiency does not inhibit caspase-1 activation induced by LPS plus ATP or nigericin, MSU, curdlan, poly-IC and poly (dA:dT), which is dependent on NLRP3, AIM2, NLRC4 or NLRP3 inflammasomes. Therefore, NLRC5-mediated inflammasomes activation may be induced by unknown pathogenic factors or endogenous activators, and its activation may be cell-type specific (42). Overall, it remains to be seen whether NLRC5 is an activating receptor of inflammasomes.

#### NLRC5 Methylation

DNA methylation usually occurs on cytosine residues of cytosine-guanosine (CpG) dinucleotides (46). Changes in CpG methylation induce changes in gene expression that can lead to changes in protein and metabolite levels, which are associated with many complex disorders (47). CpG sites in NLRC5 (cg07839457, cg16411857, cg08159663, cg00218406, and cg08099136), associated with one or several of the seven proteins of the immune system (CD48, CD163, CXCL10, CXCL11, LAG3, FCGR3B, and B2M), form a network involving the methylation of NLRC5 (47). NLRC5 increases the expression of the B2M protein of MHC class I; indirect links have been found between the five CpG sites and the associated proteins through various interleukins (IL-6 and IL-10), major histocompatibility complexes [HLA-A, HLA-ABC, MHC class I, and interferon regulatory factor 3 (IRF3)], and NF-κB (47). Moreover, it has been found that the methylation of NLRC5 is linked to numerous diseases. For example, NLRC5 methylation at cg07839457 is associated with BMI and obesity in Africans (48), whereas cg07839457 and cg16411857 methylation are associated with HIV infection (49). Moreover, NLRC5 methylation has been associated with lupus (46) and rheumatoid arthritis (50). In melanoma and bladder cancer, Low methylation of the NLRC5 promoter is associated with higher survival rate (51).Therefore, the NLRC5 methylation network plays a role in diseases and immunity, and its relationship with CNS diseases and specific disease mechanisms needs to be further elucidated.

#### NLRC5-Mediated Pathways

##### PI3K/AKT Signaling Pathway

**Figure 3:**
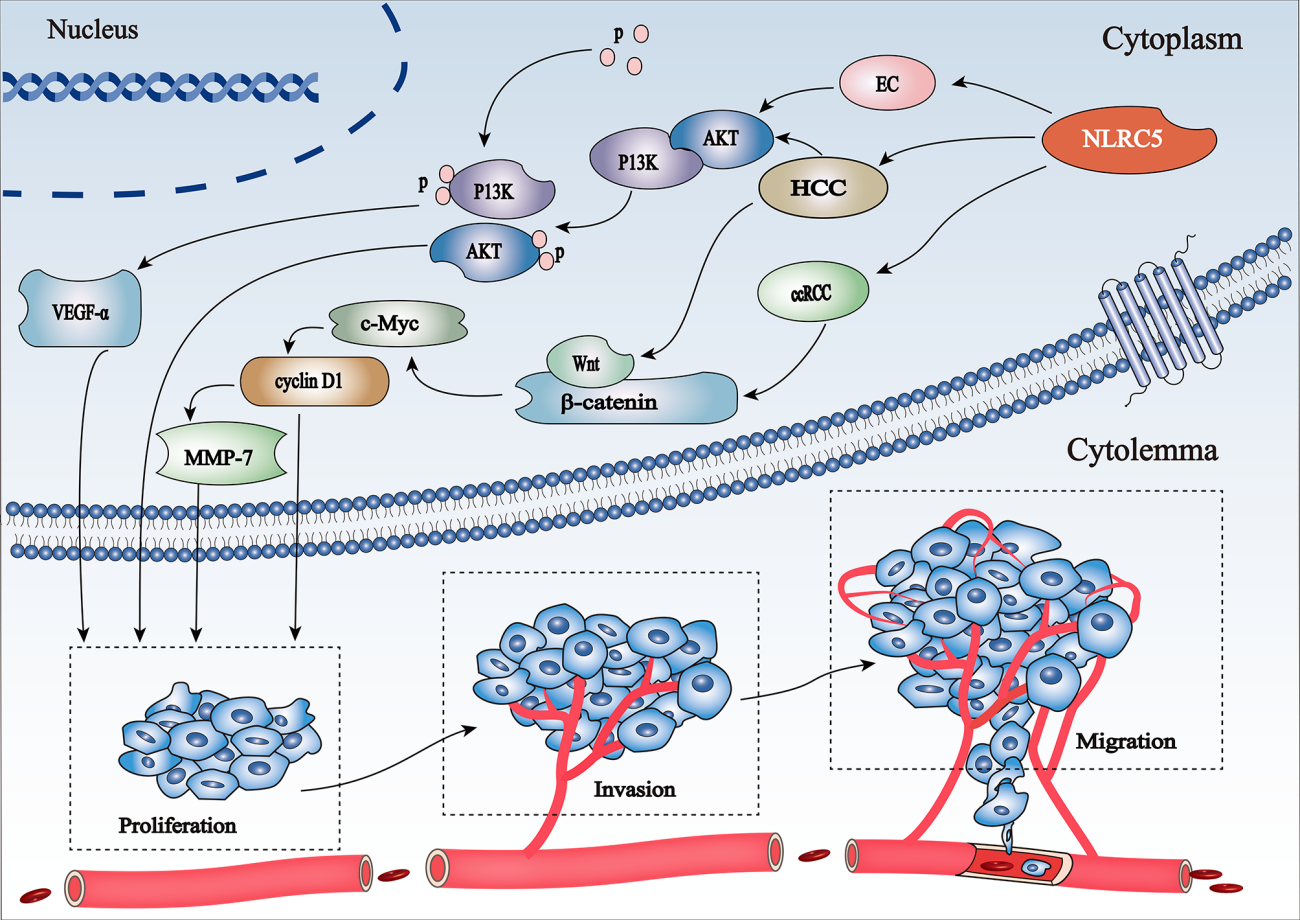
Schematic representation of regulation of the PI3K/Akt and Wnt/β-catenin pathways involved in cancer progression by NLRC5 (1) NLRC5 promotes HCC progression *via* the AKT/VEGF-A signaling pathway. (2) NLRC5 mediates cell proliferation, invasion, and migration in HCC and ccRCC by activating c-Myc and cyclin D1, the key target genes of the Wnt/β-catenin signaling pathway.

NLRC5 exerts different effects in different diseases by promoting and/or inhibiting the PI3K/AKT signaling pathway. Activation of the PI3K/AKT signaling pathway is a marker of cancer progression (52). NLRC5 promotes AN3CA cell migration and invasion by activating the PI3K/AKT signaling pathway in endometrial cancer; however, it can be inhibited by LY294002, a specific inhibitor of the PI3K/AKT signaling pathway (53). Moreover, integrin alpha x (ITGAX) stimulates cancer angiogenesis through PI3K/AKT signaling-mediated VEGFR2/VEGF-A overexpression in blood vessel endothelial cells in hepatocellular carcinoma (HCC) (54). There is a positive correlation between NLRC5 and VEGF-A expression, which also coordinates the activation of the PI3K/AKT signaling pathway (55), demonstrating that NLRC5 promotes HCC progression *via* the AKT/VEGF-A signaling pathway (**Figure 3**). Furthermore, Feng et al. found that knockdown of NLRC5 efficiently inhibited H/R-induced oxidative stress and apoptosis in HK-2 cells and attenuated renal I/R injury *in vitro* through the activation of the PI3K/AKT signaling pathway (56).

##### Wnt/β-Catenin Signaling Pathway

Wnt/β-catenin is associated with the proliferation, invasion, and migration of tumor cells (57). NLRC5 activates β-catenin transcription and translation by stimulating the Wnt/β-catenin signaling pathway, thereby promoting tumor development and progression. The oncogene c-Myc (58) and cell cycle regulator cyclin D1 (59) are key target genes of the Wnt/β-catenin signaling pathway. NLRC5 mediates cell proliferation, migration, and invasion by regulating the Wnt/β-catenin signaling pathway in clear cell renal cell carcinoma (ccRCC) (60) (**Figure 3**). However, knockdown of NLRC5 can weaken the expression of c-Myc and cyclin D1, thereby inhibiting the growth of tumor cells (60). Moreover, upregulation of NLRC5 not only positively correlates with the increase in β-catenin, but also coordinates the activation of the downstream Wnt/β-catenin signaling pathway in HCC (61).

##### Other Signaling Pathways

In addition to the aforementioned pathways, NLRC5 is involved in the regulation of the TLR4/MyD88/NF-κB, JAK2/STAT3, and TGF-β1/Smad signaling pathways, and plays a role in hepatic fibrosis, malignant tumors, ethanol-induced hepatic injury and other diseases, as shown in **Table 2**.

**Table 2:** Role of NLRC5 in common signaling pathways.

Cell/tissue	Gene expression regulation	Signaling pathway	Function	Reference
Microglial cells	Downregulated	miRNA-34a/NLRC5/NF-κB axis	Microglial activation	(6)
Cortical neuron, Brain microvessels	Upregulated	NLRC5/NLRP3/caspase-1/IL-1β	Protective effect on ZIKV and WNV infection	(7–9, 62)
Hippocampal neuron	Upregulated	NLRC5/MHC I	Promote neuronal development,	(10)
Hippocampal neuron	Upregulated	NLRC5/Nrf2/HO-1	Attenuating cerebral I/R injury induced by OGD/R	(11)
PC12 cells	Upregulated	NLRC5/TLR4/MyD88/NF-κB	Reduce inflammatory response, oxidative damage, and apoptosis induced by OGD/R	(12)
Immunocompetent mouse glioma models	Upregulated	IFN-γ/STAT1/NLRC5	Increase the antitumor immune response and extended survival	(15)
Glioma cells	Downregulated	LncRNA SCAMP1/SCAMP1/miR-499a-5p/LMX1A/NLRC5/Wnt/β-catenin	Suppress malignant biological behaviors	(13)
Cardiac fibroblasts	Upregulated	miR-214-3p/NLRC5 axis	Promote fibroblast proliferation and fibroblast-to-myofibroblast transition	(63)
Hepatic stellate cells	Upregulated	TGF-β1/NF-κB/NLRC5 TGF-β1/Smad/NLRC5	Promote hepatic fibrosis	(64)
Liver	Upregulated	LncRNA MEG3/miR-let-7c-5p/NLRC5	Promote EtOH-induced hepatic injury	(65)
Hepatocellular carcinoma cells	Upregulated	NLRC5/PI3K/AKT/VEGF-A	Promotes hepatocellular carcinoma cell progression	(55)
Hepatocellular carcinoma cells	Upregulated	NLRC5/Wnt/β-catenin	Promotes hepatocellular carcinoma cell progression	(61)
HK-2	Downregulated	NLRC5/PI3K/AKT	Inhibits H/R-induced oxidative stress and apoptosis	(56)
Renal fibroblasts	Downregulated	NLRC5/TGF-β1/Smad	Inhibits renal fibroblast activation and fibrogenesis	(66)
Macrophages	Downregulated	JAK2/STAT3/NLRC5	Promotes the secretion of IL-6 and TNF-α	(67)
Macrophages	Downregulated	Tim-3/STAT1/NLRC5/MHC I	Promotes immune evasion	(68)

## NLRC5 and the CNS

### Neuron Development

Previous studies have shown that MHC I is involved in regulating synaptic plasticity in the lateral geniculate nucleus during development, neurite outgrowth, and polarization in young cultured hippocampal neurons. Moreover, it is critical for hippocampal-dependent memory by inhibiting NMDAR function (69–71), which is of great significance to the development of neurons. Li et al. suggested that the expression of endogenous *NLRC5* mRNA and MHC I is correlated with hippocampal development (10). MHC I transcription may be regulated by NLRC5 in hippocampal neurons. The specific regulatory mechanism involves transport of NLRC5 from the cytoplasm to the nucleus and induction of the activation of MHC I transcription by binding to RFX5 and RFXANK of X1 box, CREB of X2 box, and NFY complexes (10). According to double immunostaining analysis, NLRC5 and MHC I are co-localized in hippocampal neurons, and *Nlrc5* ^-/-^ mouse hippocampal MHC I levels are significantly decreased (10), further suggesting that NLRC5 may play a vital role in regulating neuron development by upregulation of MHC I in hippocampal neurons. However, no relevant functional experiments have revealed how NLRC5 affects hippocampal neuronal function changes.

### CNS Disease

#### CNSI

The BBB is the interface that separates neural tissue from circulating blood and maintains a safe and homeostatic milieu for proper neuronal function and synaptic transmission (72). As a key part of the BBB, the brain endothelial cells can directly contact the blood and elicit bacteria–host interactions that assist bacterial pathogens, such as *E. coli*, *Streptococcus pneumoniae*, *Meningitis neisseria*, and *S. aureus*, in invading the brain (73). NLRC5 is widely expressed in cerebral endothelial cells and brain pericytes and is regulated by inflammatory mediators (2, 26–28); for example, IFN-γ, TNF-α, and IL-1β upregulate the expression of NLRC5 in brain pericytes (27).However, the role of NLRC5 in the subsequent inflammatory response and its mechanism needs to be further studied.

CNS complications in people infected with HIV are collectively termed as neuroHIV, and its pathological mechanism involves aggravation of cellular oxidative stress, disturbance of energy metabolism, immune activation, inflammation, and neuronal damage (6, 74). Many reports have confirmed both protein and mRNA expression of HIV-1 Tat in the brains of HIV-1-infected individuals. HIV-1 Tat can induce neuroinflammation by activating glial cells and modulating various signaling pathways to impact microglial function, thus exerting neurotoxic effects on many CNS cells (6). miRNA-34a is abundant in the brain and is upregulated in numerous neurological diseases and aging-related diseases (75). Periyasamy et al. showed that HIV-1 Tat-mediated upregulation of miRNA-34a targets the 3’-UTR of NLRC5 in mouse primary microglial cells and that the downregulated NLRC5 inflammasome negatively regulates the NF-κB p65 signaling axis *via* modulation of the phosphorylated IKK complex, leading to increased expression of pro-inflammatory cytokines such as IL-1β and IL-6 and the consequent microglial activation (6). In summary, the miRNA-34a/NLRC5/NF-κB signaling pathway may be an entry point for the treatment of HIV-1 Tat-mediated microglial inflammation.

Neurotropic viruses can invade the CNS *via* various routes and irreversibly disrupt the complex structural and functional architecture of the CNS, including retrograde axonal transport along motor and olfactory neurons and hematogenous spread across the BBB *via* direct infection of endothelial cells (76). Zika virus (ZIKV) and West Nile virus (WNV) have significant neuroinvasive characteristics and are regarded as neurotropic (7). High ZIKV replication was observed as early as 12 h and peaked at 48 h after infection, similar to the expression of NLRC5 mRNA in primary cortical neurons of mice (8). Leda et al. evaluated the impact of ZIKV infection on brain microvasculature and the BBB by evaluating the transcriptomic profile of brain microvessels *via* high-throughput RNA sequencing, and the analysis revealed upregulation of NLRC5 (7). Moreover, NLRC5 expression was upregulated in the mouse brain after WNV NY99 infection (9), suggesting that neurotropic virus invading the CNS could induce the expression of NLRC5 in the brain of mice. Previous studies have confirmed that NLRP3 activates caspase-1 by recruiting ASC to form inflammasomes, resulting in the proteolytic cleavage of pro-IL-1β into mature IL-1β. The NLRP3 inflammasome pathway and IL-1β signaling are key factors controlling WNV infection and immunity in the CNS (62). However, the biological function of NLRC5 in the CNS against viral infection and whether it cooperates with activation of the NLRP3 inflammasome remain unclear and need to be further studied.

#### Cerebral I/R Injury

Increasing evidence shows that NLR proteins are involved in the pathogenesis of cerebral I/R injury (77, 78). The production of reactive oxygen species (ROS) and subsequent oxidative stress, inflammation, and apoptosis are the core processes of cerebral I/R injury (79–81). Previous studies have confirmed that NLRC5 may play a key role in alleviating liver and renal I/R injury (56, 82). Li et al. showed that the mRNA and protein levels of NLRC5 are significantly decreased in oxygen-glucose deprivation (OGD)/reoxygenation (R)-induced neurons (11). Overexpression of NLRC5 has been shown to cause a significant increase in cell viability and increased expression of bcl-2, nuclear factor erythroid 2-related factor 2 (Nrf2), heme oxygenase-1 (HO-1), NAD(P)H:quinone oxidoreductase 1 (NQO-1), glutathione peroxidase 3 (GPx-3), and downstream genes of the Nrf2/HO-1 pathway, as well as decreased expression of Bax and ROS (11). In conclusion, these results suggest that the protective effects of NLRC5 are mediated by the Nrf2/HO-1 pathway. Thus, NLRC5 may serve as an effective target for the treatment of cerebral I/R injury. Furthermore, Zhang et al. found that the expression of *NLRC5* in neonates with cerebral ischemia and OGD/R-induced PC12 cells was significantly reduced (12). However, NLRC5 overexpression was found to suppress the levels of inflammatory cytokines such as TNF-α, IL-6, IL-1β, ROS, malondialdehyde (MDA), Bax, and caspase-3, as well as apoptosis. Moreover, it inhibits the levels of TLR4, MyD88, and NF-κB p-p65 and upregulates the expression of superoxide dismutase (SOD) and bcl-2 (12). Therefore, NLRC5 alleviates inflammation, oxidative damage, and apoptosis in PC12 cells under OGD/R conditions by suppressing the activation of the TLR4/MyD88/NF-κB pathway. Collectively, these results indicate that NLRC5 plays a neuroprotective role in cerebral I/R injury through different approaches, and it is expected to be an effective target for the treatment of cerebral I/R injury.

#### Glioma

Glioblastoma (GBM) is the most common and lethal type of tumor in the CNS. *NLRC5*, an IFN-related gene, has been shown to be associated with overall survival of GBM patients (14). IFN-γ is mainly produced by T and NK cells It can induce a positive feedback in the STAT1 pathway that triggers the expression and activation of STAT1 and other downstream genes, such as *NLRC5*, *CIITA*, and *TAP1*, and increases the immune response in the tumor microenvironment (5, 15). *In vivo* genetic knockdown of the proto-oncogene Fyn can induce changes in IFN-γ secretion, promote the overexpression of STAT1 and its downstream genes, and further increase the antitumor immune response in immunocompetent mouse glioma models, which significantly extends survival (15). In addition, upregulation of the lncRNA secretory carrier membrane protein 1 (SCAMP1), which functions as an oncogene in glioma cells, significantly promotes glioma cell proliferation, migration, and invasion and inhibits apoptosis (13). In contrast, miR-499a-5p exerts a tumor-suppressive function in glioma cells (13). SCAMP1 can increase LIM homeobox transcription factor 1 alpha (LMX1A) levels by negatively regulating miR-499a-5p expression, following LMX1A-mediated transcriptional activation of NLRC5, and can promote malignant biological behavior of glioma cells by attenuating the activity of the Wnt/β-catenin signaling pathway (13). This suggests that NLRC5 plays a regulatory role in the development of glioma and is related to IFN signaling and the SCAMP1/miR-499a-5p/LMX1A/NLRC5 pathway.

#### MS

Multiple sclerosis (MS) is an autoimmune disease of the CNS, associated with inappropriate activation of lymphocytes, hyperinflammatory responses, demyelination, and neuronal damage (83). NLRs, as inflammatory regulators expressed in various cells in the CNS, mediate a variety of signaling pathways and play a role in the pathogenesis of MS (83). Falcão et al. performed single-cell transcriptomic analysis of oligodendrocyte lineage cells from the spinal cord of mice with experimental autoimmune encephalomyelitis (EAE) and showed that EAE-specific oligodendrocyte lineage populations express genes involved in antigen processing and presentation *via* MHC I and MHC II and genes involved in immunoprotection (16). Transcription factors (such as NLRC5, a transactivator of MHC I) are expressed in all oligodendrocyte lineage cells. These factors are induced by the IFN-γ pathway and can be used as MS-specific markers (16). Overall, as the transactivator of MHC I, the role of NLRC5 in MS and its mechanism are worthy of further exploration.

#### Epilepsy

Epilepsy is a group of diseases characterized by periodic seizures and unpredictable occurrences caused by abnormal synchronous activity of neurons. Theiler’s murine encephalomyelitis virus (TMEV) infection induces a well-characterized experimental model of epilepsy, resulting in increased expression of *NLRC5* and *MHC I* (H2-Kb) mRNAs and significantly decreased expression of IFN-I and pro-inflammatory mediators in normal mouse brains (17). It was found that infected *Nlrc5* ^-/-^ mice had significantly fewer seizures, lower sickness scores, weight loss, and neuroinflammation than wild-type mice (17). NLRC5 deficiency can reduce epileptic seizures caused by immune abnormalities in TMEV-infected mice, suggesting that NLRC5 can enhance the inflammatory response, regulate antiviral immunity, and promote the occurrence of epileptic seizures. Thus, inhibiting the acute elevation of NLRC5 levels or its downstream signaling molecules, such as IL-1β, is a promising therapeutic strategy for epilepsy. In addition, other members of the NLR family, such as NLRP1, have previously been implicated in epilepsy and were found to contribute to neuronal death and chronic seizure activity in the amygdala kindling-induced rat model of epilepsy (84). Since NLRC5 can interact with other NLRs (such as NLRP3) and form inflammasomes, further study of the synergistic effect of NLRC5 and other inflammasomes on epileptic seizures is also necessary.

#### SCZ and BD

SCZ and BD are highly heritable psychiatric disorders with overlapping susceptibility loci and symptomatology, such as impulsive and risk-taking behaviors (18, 19). NLRC5 is upregulated in SCZ and is located in the genetic signaling regions of BD and SCZ (18). Furthermore, Pacifico et al. reported the first transcriptome sequencing of the postmortem human dorsal striatum, their data showed that NLRC5 was differentially expressed between bipolar (n = 18) and control (n = 17) subjects (19). However, whether the highly expressed nlrc5 is involved in the development of SCZ and BD remains to be elucidated.

## Conclusions

MHC I antigen presentation, MHC I-regulated of neuronal development, activation of the NF-κB signaling pathway, and activation of inflammasomes are important components of the immune response in nervous system development and nervous system diseases. NLRC5, the largest member of the NLR family, participates in the pathophysiological process of the CNS by regulating multiple pathways such as NF-κB, IFN-I, and inflammatory signaling pathways and MHC I gene expression. On the one hand, it can promote the development of neurons, has a neuroprotective effect against HIV and other viral encephalitis and brain I/R injury, and can also enhance the immune response against cerebral tumors. However, it may also play a negative role in the occurrence of neuropsychiatric diseases such as epilepsy, SCZ, and BD and may promote the malignant biological behavior of brain tumor cells. In summary, the findings from studies on NLRC5 provide new insights into the pathogenesis and treatment of various neurological diseases.

Although progress has been made in the research into the effect of NLRC5 in the CNS, its role and application still need to be further explored and expanded. For example, CNSI by bacteria is the most common and severe infectious disease in children, which can lead to serious neurological sequelae. However, there are few studies on NLRC5 and bacterial infections of the CNS. Based on its unique role in innate immunity, we can further focus on the immune signal regulation pathway in bacterial CNSI, which is conducive to providing a new theoretical basis for the targeted therapy of clinically relevant diseases. In addition, NLRC5 upregulates MHC I in neurons, and its downstream target, NF-κB, is closely related to synaptic plasticity. Therefore, in addition to the molecular mechanism, the effects of NLRC5 on synaptic function and neural information transmission can be studied in combination with electrophysiological and other functional experiments to explore its mechanism in neural development and neuropsychiatric diseases.

## Author Contributions

LQL, LZ, CJ, and DM conceived the idea of this review. CJ and LZ performed literature searching and drafted the manuscript. LJL, AW, LT, YR, PH, and JX created the figures. All authors critically reviewed and edited the content of this manuscript. All authors contributed to the article and approved the submitted version.

## Funding

This work was supported by National Natural Science Foundation of China (No. 81873762 to LQL and No. 81501039 to LJL), and the Program of Hunan Provincial Department of Science and Technology, China (No. 2018SK2069 to LQL), and the Natural Science Foundation of Hunan Province, China (No. 2019JJ50876 to JX), and the Program from Health Commission of Hunan Province, China (No. B2018-0311 to LQL).

## Conflict of Interest

The authors declare that the research was conducted in the absence of any commercial or financial relationships that could be construed as a potential conflict of interest.

## Abbreviations

ASC, Apoptosis-associated speck-like protein containing CARD; ATF, Activating transcription; BBB, blood-brain barrier; BD, bipolar disorder; BIR, Baculovirus inhibitor domain; CARD, Containing caspase recruitment domain; CBP, CREB binding protein; ccRCC, Clear cell renal cell carcinoma; CIITA, Class II transactivator; CLRs, C-type lectin receptors; CNS, Central nervous system; CNSI, Central nervous system infection; CpG, Cytosine-guanosine dinucleotides; CREB, cAMP response element binding protein; DAMPs, Damage-associated molecular patterns; EAE, Experimental autoimmune encephalomyelitis; GAS, Gamma interferon activation site; GBM, Glioblastoma; GPx-3, Glutathione peroxidase 3; HCC, Hepatocellular carcinoma; HO-1, Heme oxygenase-1; IFN-1, Type I interferon; I/R, Ischemia/reperfusion; ITGAX, Integrin alpha x; LMX1A, LIM homeobox transcription factor 1, alpha; LRRs, Leucine-rich repeats; MDA, Malondialdehyde; MS, Multiple sclerosis; MHC, major histocompatibility complex; NF-κB, Nuclear factor-κB; NFY, Nuclear factor-Y protein; NLRs, NOD-like receptors; NLRC5, NLR family, CARD containing 5; NLRP3, NLR family pyrin domain containing 3; NLS, Nuclear localization signal; NODs, Nucleotide oligomerization domain; NQO-1, NAD(P)H,quinone oxidoreductase 1; Nrf2, Nuclear factor erythroid 2-related factor 2; NTP, nucleoside triphosphate; OGD, Oxygen-glucose deprivation; PAMPs, Pathogen-associated molecular patterns; PRRs, Pattern recognition receptors; PYD, Pyrin domain; RFX, Regulatory factor X; RLRs, Retinoic acid-inducible gene-I-like receptors; ROS, oxygen species; TLRs, Toll-like receptors; SCAMP1, Secretory carrier membrane protein 1; SCZ, Schizophrenia; TMEV, Theiler’s murine encephalomyelitis virus; VSV, Vesicular stomatitis virus; SOD, Superoxide dismutase; STAT1, Signal transducers and activators of transcription-1; WNV, West Nile virus; ZIKV, Zika virus.

## References

[1] Wu Y Shi T Li J. NLRC5: A Paradigm for NLRs in Immunological and Inflammatory Reaction. Cancer Lett (2019) 451:92–9. 10.1016/j.canlet.2019.03.005 30867141

[2] Kong X Yuan Z Cheng J. The Function of NOD-like Receptors in Central Nervous System Diseases. J Neurosci Res (2017) 95(8):1565–73. 10.1002/jnr.24004 28029680

[3] Wang JQ Liu YR Xia Q Chen RN Liang J Xia QR. Emerging Roles for NLRC5 in Immune Diseases. Front Pharmacol (2019) 10:1352. 10.3389/fphar.2019.01352 31824312 PMC6880621

[4] Cao L Wu XM Hu YW Xue NN Nie P Chang MX. The Discrepancy Function of NLRC5 Isoforms in Antiviral and Antibacterial Immune Responses. Dev Comp Immunol (2018) 84:153–63. 10.1016/j.dci.2018.02.013 29454830

[5] Qiu L Ma T Chang G Liu X Guo X Xu L. Expression Patterns of NLRC5 and Key Genes in the STAT1 Pathway Following Infection With Salmonella pullorum. Gene (2017) 597:23–9. 10.1016/j.gene.2016.10.026 27771450

[6] Periyasamy P Thangaraj A Bendi VS Buch S. HIV-1 Tat-mediated Microglial Inflammation Involves a Novel miRNA-34a-NLRC5-NfκB Signaling Axis. Brain Behav Immun (2019) 80:227–37. 10.1016/j.bbi.2019.03.011 PMC6660398 30872089

[7] Leda AR Bertrand L Andras IE El-Hage N Nair M Toborek M. Selective Disruption of the Blood-Brain Barrier by Zika Virus. Front Microbiol (2019) 10:2158. 10.3389/fmicb.2019.02158 31620112 PMC6759472

[8] Azouz F Arora K Krause K Nerurkar VR Kumar M. Integrated MicroRNA and Mrna Profiling in Zika Virus-Infected Neurons. Viruses (2019) 11(2):162. 10.3390/v11020162 PMC6410042 30781519

[9] Kumar M Belcaid M Nerurkar VR. Identification of Host Genes Leading to West Nile Virus Encephalitis in Mice Brain Using RNA-seq Analysis. Sci Rep (2016) 6:26350. 10.1038/srep26350 27211830 PMC4876452

[10] Li P Shen Y Cui P Hu Y Zhang Y Miao F. Neuronal NLRC5 Regulates MHC Class I Expression in Neuro-2a Cells and Also During Hippocampal Development. J Neurochem (2020) 152(2):182–94. 10.1111/jnc.14876 31549732

[11] Li L Yu M Pang H Chen L Liu J Hou S. NLRC5 Protects Neurons From Oxygen-Glucose Deprivation-Induced Injury Through Activating the Nrf2/HO-1 Pathway. J Recept Signal Transduct Res (2021) 41(1):53–8. 10.1080/10799893.2020.1786840 32605461

[12] Zhang Z Sun Y Chen X. NLRC5 Alleviated OGD/R-Induced PC12-Cell Injury by Inhibiting Activation of the TLR4/Myd88/NF-κb Pathway. J Int Med Res (2020) 48(8):300060520940455. 10.1177/0300060520940455 32790491 PMC7427022

[13] Zong Z Song Y Xue Y Ruan X Liu X Yang C. Knockdown of LncRNA SCAMP1 Suppressed Malignant Biological Behaviours of Glioma Cells *via* Modulating miR-499a-5p/LMX1A/NLRC5 Pathway. J Cell Mol Med (2019) 23(8):5048–62. 10.1111/jcmm.14362 PMC6653555 31207033

[14] Zhu C Zou C Guan G Guo Q Yan Z Liu T. Development and Validation of an Interferon Signature Predicting Prognosis and Treatment Response for Glioblastoma. Oncoimmunology (2019) 8(9):e1621677. 10.1080/2162402X.2019.1621677 31428519 PMC6685507

[15] Comba A Dunn PJ Argento AE Kadiyala P Ventosa M Zamler DB. The Proto-Oncogene Fyn Inhibits the Anti-Glioblastoma Immune Response [Preprint] (2019). 10.1101/608505

[16] Falcão AM van Bruggen D Marques S Meijer M Jäkel S Agirre E. Disease-Specific Oligodendrocyte Lineage Cells Arise in Multiple Sclerosis. Nat Med (2018) 24(12):1837–44. 10.1038/s41591-018-0236-y PMC6544508 30420755

[17] Bijalwan M. Pathogenesis of Theiler’s Murine Encephalomyelitis Virus (TMEV) in an Experimental Model of Epilepsy. College Station, Texas, USA: Texas A&M University (2017).

[18] Bergen SE O’Dushlaine CT Ripke S Lee PH Ruderfer DM Akterin S. Genome-Wide Association Study in a Swedish Population Yields Support for Greater CNV and MHC Involvement in Schizophrenia Compared With Bipolar Disorder. Mol Psychiatry (2012) 17(9):880–6. 10.1038/mp.2012.73 PMC3724337 22688191

[19] Pacifico R Davis RL. Transcriptome Sequencing Implicates Dorsal Striatum-Specific Gene Network, Immune Response and Energy Metabolism Pathways in Bipolar Disorder. Mol Psychiatry (2017) 22(3):441–9. 10.1038/mp.2016.94 27350034

[20] Yao Y Qian Y. Expression Regulation and Function of NLRC5. Protein Cell (2013) 4(3):168–75. 10.1007/s13238-012-2109-3 PMC4875496 23483478

[21] Tang F Xu Y Zhao B. NLRC5: New Cancer Buster? Mol Biol Rep (2020) 47(3):2265–77. 10.1007/s11033-020-05253-5 31925644

[22] Gutte PG Jurt S Grütter MG Zerbe O. Unusual Structural Features Revealed by the Solution NMR Structure of the NLRC5 Caspase Recruitment Domain. Biochemistry (2014) 53(19):3106–17. 10.1021/bi500177x 24815518

[23] Jongsma MLM Guarda G Spaapen RM. The Regulatory Network Behind MHC Class I Expression. Mol Immunol (2019) 113:16–21. 10.1016/j.molimm.2017.12.005 29224918

[24] Mótyán JA Bagossi P Benkő S Tőzsér J. A Molecular Model of the Full-Length Human NOD-like Receptor Family CARD Domain Containing 5 (NLRC5) Protein. BMC Bioinf (2013) 14:275. 10.1186/1471-2105-14-275 PMC3848420 24044430

[25] Kuenzel S Till A Winkler M Häsler R Lipinski S Jung S. The Nucleotide-Binding Oligomerization Domain-Like Receptor NLRC5 is Involved in IFN-Dependent Antiviral Immune Responses. J Immunol (2010) 184(4):1990–2000. 10.4049/jimmunol.0900557 20061403

[26] Lénárt N Brough D Dénes Á. Inflammasomes Link Vascular Disease With Neuroinflammation and Brain Disorders. J Cereb Blood Flow Metab (2016) 36(10):1668–85. 10.1177/0271678X16662043 PMC5076791 27486046

[27] Nyúl-Tóth Á Kozma M Nagyőszi P Nagy K Fazakas C Haskó J. Expression of Pattern Recognition Receptors and Activation of the Non-Canonical Inflammasome Pathway in Brain Pericytes. Brain Behav Immun (2017) 64:220–31. 10.1016/j.bbi.2017.04.010 28432035

[28] Nagyőszi P Nyúl-Tóth Á Fazakas C Wilhelm I Kozma M Molnár J. Regulation of NOD-Like Receptors and Inflammasome Activation in Cerebral Endothelial Cells. J Neurochem (2015) 135(3):551–64. 10.1111/jnc.13197 26083549

[29] Meissner TB Li A Biswas A Lee KH Liu YJ Bayir E. NLR Family Member NLRC5 Is a Transcriptional Regulator of MHC Class I Genes. Proc Natl Acad Sci U S A (2010) 107(31):13794–9. 10.1073/pnas.1008684107 PMC2922274 20639463

[30] Meffert MK Chang JM Wiltgen BJ Fanselow MS Baltimore D. NF-kappa B Functions in Synaptic Signaling and Behavior. Nat Neurosci (2003) 6(10):1072–8. 10.1038/nn1110 12947408

[31] Robison AJ Nestler EJ. Transcriptional and Epigenetic Mechanisms of Addiction. Nat Rev Neurosci (2011) 12(11):623–37. 10.1038/nrn3111 PMC3272277 21989194

[32] Cui J Zhu L Xia X Wang HY Legras X Hong J. NLRC5 Negatively Regulates the NF-kappaB and Type I Interferon Signaling Pathways. Cell (2010) 141(3):483–96. 10.1016/j.cell.2010.03.040 PMC3150216 20434986

[33] Benko S Magalhaes JG Philpott DJ Girardin SE. NLRC5 Limits the Activation of Inflammatory Pathways. J Immunol (2010) 185(3):1681–91. 10.4049/jimmunol.0903900 20610642

[34] Neerincx A Lautz K Menning M Kremmer E Zigrino P Hösel M. A Role for the Human Nucleotide-Binding Domain, Leucine-Rich Repeat-Containing Family Member NLRC5 in Antiviral Responses. J Biol Chem (2010) 285(34):26223–32. 10.1074/jbc.M110.109736 PMC2924034 20538593

[35] Tong Y Cui J Li Q Zou J Wang HY Wang RF. Enhanced TLR-induced Nf-κb Signaling and Type I Interferon Responses in NLRC5 Deficient Mice. Cell Res (2012) 22(5):822–35. 10.1038/cr.2012.53 PMC3343662 22473004

[36] Fekete T Bencze D Szabo A Csoma E Biro T Bacsi A. Regulatory NLRs Control the RLR-Mediated Type I Interferon and Inflammatory Responses in Human Dendritic Cells. Front Immunol (2018) 9:2314. 10.3389/fimmu.2018.02314 30344524 PMC6182093

[37] Robbins GR Truax AD Davis BK Zhang L Brickey WJ Ting JP. Regulation of Class I Major Histocompatibility Complex (MHC) by Nucleotide-Binding Domain, Leucine-Rich Repeat-Containing (NLR) Proteins. J Biol Chem (2012) 287(29):24294–303. 10.1074/jbc.M112.364604 PMC3397855 22645137

[38] Hu Z Chai J. Structural Mechanisms in NLR Inflammasome Assembly and Signaling. Curr Top Microbiol Immunol (2016) 397:23–42. 10.1007/978-3-319-41171-2_2 27460803

[39] Yu JW Lee MS. Mitochondria and the NLRP3 Inflammasome: Physiological and Pathological Relevance. Arch Pharm Res (2016) 39(11):1503–18. 10.1007/s12272-016-0827-4 27600432

[40] Gordon R Albornoz EA Christie DC Langley MR Kumar V Mantovani S. Inflammasome Inhibition Prevents α-Synuclein Pathology and Dopaminergic Neurodegeneration in Mice. Sci Transl Med (2018) 10(465):eaah4066. 10.1126/scitranslmed.aah4066 30381407 PMC6483075

[41] Davis BK Roberts RA Huang MT Willingham SB Conti BJ Brickey WJ. Cutting Edge: NLRC5-Dependent Activation of the Inflammasome. J Immunol (2011) 186(3):1333–7. 10.4049/jimmunol.1003111 PMC3669680 21191067

[42] Kumar H Pandey S Zou J Kumagai Y Takahashi K Akira S. NLRC5 Deficiency Does Not Influence Cytokine Induction by Virus and Bacteria Infections. J Immunol (2011) 186(2):994–1000. 10.4049/jimmunol.1002094 21148033

[43] Yao Y Wang Y Chen F Huang Y Zhu S Leng Q. NLRC5 Regulates MHC Class I Antigen Presentation in Host Defense Against Intracellular Pathogens. Cell Res (2012) 22(5):836–47. 10.1038/cr.2012.56 PMC3346158 22491475

[44] Labbé K Saleh M. Cell Death in the Host Response to Infection. Cell Death Differ (2008) 15(9):1339–49. 10.1038/cdd.2008.91 18566602

[45] Fink SL Bergsbaken T Cookson BT. Anthrax Lethal Toxin and Salmonella Elicit the Common Cell Death Pathway of Caspase-1-Dependent Pyroptosis via Distinct Mechanisms. Proc Natl Acad Sci U S A (2008) 105(11):4312–7. 10.1073/pnas.0707370105 PMC2393760 18337499

[46] Yeung KS Chung BH Choufani S Mok MY Wong WL Mak CC. Genome-Wide DNA Methylation Analysis of Chinese Patients With Systemic Lupus Erythematosus Identified Hypomethylation in Genes Related to the Type I Interferon Pathway. PLoS One (2017) 12(1):e0169553. 10.1371/journal.pone.0169553 28085900 PMC5234836

[47] Zaghlool SB Kühnel B Elhadad MA Kader S Halama A Thareja G. Epigenetics Meets Proteomics in an Epigenome-Wide Association Study With Circulating Blood Plasma Protein Traits. Nat Commun (2020) 11(1):15. 10.1038/s41467-019-13831-w 31900413 PMC6941977

[48] Meeks KAC Henneman P Venema A Burr T Galbete C Danquah I. An Epigenome-Wide Association Study in Whole Blood of Measures of Adiposity Among Ghanaians: The RODAM Study. Clin Epigenet (2017) 9:103. 10.1186/s13148-017-0403-x PMC5609006 28947923

[49] Zhang X Justice AC Hu Y Wang Z Zhao H Wang G. Epigenome-Wide Differential DNA Methylation Between HIV-Infected and Uninfected Individuals. Epigenetics (2016) 11(10):750–60. 10.1080/15592294.2016.1221569 PMC5094631 27672717

[50] Zou J Lippert C Heckerman D Aryee M Listgarten J. Epigenome-Wide Association Studies Without the Need for Cell-Type Composition. Nat Methods (2014) 11(3):309–11. 10.1038/nmeth.2815 24464286

[51] Yoshihama S Roszik J Downs I Meissner TB Vijayan S Chapuy B. NLRC5/MHC Class I Transactivator is a Target for Immune Evasion in Cancer. Proc Natl Acad Sci U S A (2016) 113(21):5999–6004. 10.1073/pnas.1602069113 27162338 PMC4889388

[52] Song M Bode AM Dong Z Lee MH. AKT as a Therapeutic Target for Cancer. Cancer Res (2019) 79(6):1019–31. 10.1158/0008-5472.CAN-18-2738 30808672

[53] Fan Y Dong Z Shi Y Sun S Wei B Zhan L. NLRC5 Promotes Cell Migration and Invasion by Activating the PI3K/AKT Signaling Pathway in Endometrial Cancer. J Int Med Res (2020) 48(5):300060520925352. 10.1177/0300060520925352 32431202 PMC7241267

[54] Wang J Yang L Liang F Chen Y Yang G. Integrin Alpha X Stimulates Cancer Angiogenesis Through PI3K/Akt Signaling-Mediated VEGFR2/VEGF-A Overexpression in Blood Vessel Endothelial Cells. J Cell Biochem (2019) 120(2):1807–18. 10.1002/jcb.27480 30873824

[55] He YH Li MF Zhang XY Meng XM Huang C Li J. NLRC5 Promotes Cell Proliferation Via Regulating the AKT/VEGF-A Signaling Pathway in Hepatocellular Carcinoma. Toxicology (2016) 359-360:47–57. 10.1016/j.tox.2016.06.012 27338800

[56] Han F Gao Y Ding CG Xia XX Wang YX Xue WJ. Knockdown of NLRC5 Attenuates Renal I/R Injury *In Vitro* Through the Activation of PI3K/Akt Signaling Pathway. BioMed Pharmacother (2018) 103:222–7. 10.1016/j.biopha.2018.04.040 29655162

[57] Nusse R Clevers H. Wnt/β-Catenin Signaling, Disease, and Emerging Therapeutic Modalities. Cell (2017) 169(6):985–99. 10.1016/j.cell.2017.05.016 28575679

[58] Wang Q Zhou Y Rychahou P Harris JW Zaytseva YY Liu J. Deptor Is a Novel Target of Wnt/β-Catenin/c-Myc and Contributes to Colorectal Cancer Cell Growth. Cancer Res (2018) 78(12):3163–75. 10.1158/0008-5472.CAN-17-3107 PMC6004255 29666061

[59] Wang Z Li B Zhou L Yu S Su Z Song J. Prodigiosin Inhibits Wnt/β-Catenin Signaling and Exerts Anticancer Activity in Breast Cancer Cells. Proc Natl Acad Sci U S A (2016) 113(46):13150–5. 10.1073/pnas.1616336113 PMC5135380 27799526

[60] Wang Q Ding H He Y Li X Cheng Y Xu Q. NLRC5 Mediates Cell Proliferation, Migration, and Invasion by Regulating the Wnt/β-Catenin Signalling Pathway in Clear Cell Renal Cell Carcinoma. Cancer Lett (2019) 444:9–19. 10.1016/j.canlet.2018.11.024 30543814

[61] Peng YY He YH Chen C Xu T Li L Ni MM. NLRC5 Regulates Cell Proliferation, Migration and Invasion in Hepatocellular Carcinoma by Targeting the Wnt/β-Catenin Signaling Pathway. Cancer Lett (2016) 376(1):10–21. 10.1016/j.canlet.2016.03.006 26975630

[62] Ramos HJ Lanteri MC Blahnik G Negash A Suthar MS Brassil MM. IL-1β Signaling Promotes CNS-Intrinsic Immune Control of West Nile Virus Infection. PLoS Pathog (2012) 8(11):e1003039. 10.1371/journal.ppat.1003039 23209411 PMC3510243

[63] Yang K Shi J Hu Z Hu X. The Deficiency of miR-214-3p Exacerbates Cardiac Fibrosis *via* miR-214-3p/NLRC5 Axis. Clin Sci (Lond) (2019) 133(17):1845–56. 10.1042/CS20190203 31434695

[64] Xu T Ni MM Xing L Li XF Meng XM Huang C. NLRC5 Regulates TGF-beta1-induced Proliferation and Activation of Hepatic Stellate Cells During Hepatic Fibrosis. Int J Biochem Cell Biol (2016) 70:92–104. 10.1016/j.biocel.2015.11.010 26592197

[65] Wang Q Li M Shen Z Bu F Yu H Pan X. The Long Non-Coding RNA MEG3/Mir-Let-7c-5p Axis Regulates Ethanol-Induced Hepatic Steatosis and Apoptosis by Targeting NLRC5. Front Pharmacol (2018) 9:302. 10.3389/fphar.2018.00302 29692724 PMC5902529

[66] Wang S Zhao X Yang S Chen B Shi J. Knockdown of NLRC5 Inhibits Renal Fibroblast Activation *via* Modulating TGF-Beta1/Smad Signaling Pathway. Eur J Pharmacol (2018) 829:38–43. 10.1016/j.ejphar.2018.03.045 29608899

[67] Li L Xu T Huang C Peng Y Li J. NLRC5 Mediates Cytokine Secretion in RAW264.7 Macrophages and Modulated by the JAK2/STAT3 Pathway. Inflammation (2014) 37(3):835–47. 10.1007/s10753-013-9804-y 24445959

[68] Wang Z Li G Dou S Zhang Y Liu Y Zhang J. Tim-3 Promotes Listeria Monocytogenes Immune Evasion by Suppressing Major Histocompatibility Complex Class I. J Infect Dis (2020) 221(5):830–40. 10.1093/infdis/jiz512 31586389

[69] Lee H Brott BK Kirkby LA Adelson JD Cheng S Feller MB. Synapse Elimination and Learning Rules Co-Regulated by MHC Class I H2-Db. Nature (2014) 509(7499):195–200. 10.1038/nature13154 24695230 PMC4016165

[70] Shen Y Zhao H Li P Peng Y Cui P Miao F. MHC Class I Molecules and PirB Shape Neuronal Morphology by Affecting the Dendritic Arborization of Cortical Neurons. Neurochem Res (2019) 44(2):312–22. 10.1007/s11064-018-2676-7 30406910

[71] Fourgeaud L Davenport CM Tyler CM Cheng TT Spencer MB Boulanger LM. MHC Class I Modulates NMDA Receptor Function and AMPA Receptor Trafficking. Proc Natl Acad Sci U S A (2010) 107(51):22278–83. 10.1073/pnas.0914064107 PMC3009822 21135233

[72] Langen UH Ayloo S Gu C. Development and Cell Biology of the Blood-Brain Barrier. Annu Rev Cell Dev Biol (2019) 35:591–613. 10.1146/annurev-cellbio-100617-062608 31299172 PMC8934576

[73] Al-Obaidi MMJ Desa MNM. Mechanisms of Blood Brain Barrier Disruption by Different Types of Bacteria, and Bacterial-Host Interactions Facilitate the Bacterial Pathogen Invading the Brain. Cell Mol Neurobiol (2018) 38(7):1349–68. 10.1007/s10571-018-0609-2 PMC11481977 30117097

[74] Saylor D Dickens AM Sacktor N Haughey N Slusher B Pletnikov M. HIV-Associated Neurocognitive Disorder–Pathogenesis and Prospects for Treatment. Nat Rev Neurol (2016) 12(4):234–48. 10.1038/nrneurol.2016.27 PMC4937456 26965674

[75] Jauhari A Singh T Singh P Parmar D Yadav S. Regulation of miR-34 Family in Neuronal Development. Mol Neurobiol (2018) 55(2):936–45. 10.1007/s12035-016-0359-4 28084588

[76] Ludlow M Kortekaas J Herden C Hoffmann B Tappe D Trebst C. Neurotropic Virus Infections as the Cause of Immediate and Delayed Neuropathology. Acta Neuropathol (2016) 131(2):159–84. 10.1007/s00401-015-1511-3 PMC4713712 26659576

[77] Wang H Zhong D Chen H Jin J Liu Q Li G. NLRP3 Inflammasome Activates Interleukin-23/interleukin-17 Axis During Ischaemia-Reperfusion Injury in Cerebral Ischaemia in Mice. Life Sci (2019) 227:101–13. 10.1016/j.lfs.2019.04.031 31002919

[78] Meng C Zhang J Zhang L Wang Y Li Z Zhao J. Effects of NLRP6 in Cerebral Ischemia/Reperfusion (I/R) Injury in Rats. J Mol Neurosci (2019) 69(3):411–8. 10.1007/s12031-019-01370-4 31267316

[79] Huang Q Lou T Wang M Xue L Lu J Zhang H. Compound K Inhibits Autophagy-Mediated Apoptosis Induced by Oxygen and Glucose Deprivation/Reperfusion *via* Regulating AMPK-mTOR Pathway in Neurons. Life Sci (2020) 254:117793. 10.1016/j.lfs.2020.117793 32416164

[80] Deng Y Ma G Dong Q Sun X Liu L Miao Z. Overexpression of miR-224-3p Alleviates Apoptosis From Cerebral Ischemia Reperfusion Injury by Targeting FIP200. J Cell Biochem (2019) 120(10):17151–8. 10.1002/jcb.28975 31134677

[81] Shi YS Zhang Y Liu B Li CB Wu J Li Y. Nomilin Protects Against Cerebral Ischemia-Reperfusion Induced Neurological Deficits and Blood-Brain Barrier Disruption *via* the Nrf2 Pathway. Food Funct (2019) 10(9):5323–32. 10.1039/C9FO01481K 31389456

[82] Chen Z Ding T Ma CG. Dexmedetomidine (DEX) Protects Against Hepatic Ischemia/Reperfusion (I/R) Injury by Suppressing Inflammation and Oxidative Stress in NLRC5 Deficient Mice. Biochem Biophys Res Commun (2017) 493(2):1143–50. 10.1016/j.bbrc.2017.08.017 28784305

[83] Gharagozloo M Gris KV Mahvelati T Amrani A Lukens JR Gris D. NLR-Dependent Regulation of Inflammation in Multiple Sclerosis. Front Immunol (2017) 8:2012. 10.3389/fimmu.2017.02012 29403486 PMC5778124

[84] Tan CC Zhang JG Tan MS Chen H Meng DW Jiang T. NLRP1 Inflammasome Is Activated in Patients With Medial Temporal Lobe Epilepsy and Contributes to Neuronal Pyroptosis in Amygdala Kindling-Induced Rat Model. J Neuroinflamm (2015) 12:18. 10.1186/s12974-014-0233-0 PMC4314732 25626361

